# Measurable residual disease analysis in paediatric acute lymphoblastic leukaemia patients with ABL-class fusions

**DOI:** 10.1038/s41416-022-01806-6

**Published:** 2022-06-01

**Authors:** Nicola C. Venn, Libby Huang, Lenka Hovorková, Walter Muskovic, Marie Wong, Tamara Law, Susan L. Heatley, Seong Lin Khaw, Tom Revesz, Luciano Dalla Pozza, Peter J. Shaw, Chris Fraser, Andrew S. Moore, Siobhan Cross, Katerina Bendak, Murray D. Norris, Michelle J. Henderson, Deborah L. White, Mark J. Cowley, Toby N. Trahair, Jan Zuna, Rosemary Sutton

**Affiliations:** 1 Molecular Diagnostics, Children’s Cancer Institute, Lowy Cancer Research Centre, UNSW, Sydney, NSW Australia; 2 Department of Paediatric Haematology and Oncology, Second Faculty of Medicine, Charles University, Prague, Czech Republic; 3 CLIP—Childhood Leukaemia Investigation Prague, Prague, Czech Republic; 4 Computational Biology, Children’s Cancer Institute, Lowy Cancer Research Centre, UNSW, Sydney, NSW Australia; 5 Precision Medicine Theme, South Australian Health and Medical Research Institute, Adelaide, SA Australia; 6 School of Medicine, University of Adelaide, Adelaide, SA Australia; 7 Children’s Cancer Centre, The Royal Children’s Hospital, Melbourne, VIC Australia; 8 Department of Clinical Haematology and Oncology, Women’s and Children’s Hospital, Adelaide, SA Australia; 9 Cancer Centre for Children, The Children’s Hospital at Westmead, Sydney, NSW Australia; 10 Blood and Bone Marrow Transplant Program, Queensland Children’s Hospital, Brisbane, QLD Australia; 11 Paediatric Oncology, Queensland Children’s Hospital, Brisbane, QLD Australia; 12 Children’s Haematology/Oncology Centre Christchurch Hospital, Christchurch, New Zealand; 13 School of Women’s and Children’s Health, University of New South Wales, Sydney, NSW Australia; 14 Kids Cancer Centre, Sydney Children’s Hospital, Randwick, NSW Australia; 15 University Hospital Motol, Prague, Czech Republic

**Keywords:** Prognostic markers, Acute lymphocytic leukaemia

## Abstract

**Background:**

ABL-class fusions including *NUP214-ABL1* and *EBF1-PDGFRB* occur in high risk acute lymphoblastic leukaemia (ALL) with gene expression patterns similar to *BCR-ABL*-positive ALL. Our aim was to evaluate new DNA-based measurable residual disease (MRD) tests detecting these fusions and *IKZF1*-deletions in comparison with conventional immunoglobulin/T-cell receptor (Ig/TCR) markers.

**Methods:**

Precise genomic breakpoints were defined from targeted or whole genome next generation sequencing for ABL-fusions and *BCR-ABL1*. Quantitative PCR assays were designed and used to re-measure MRD in remission bone marrow samples previously tested using Ig/TCR markers. All MRD testing complied with EuroMRD guidelines.

**Results:**

ABL-class patients had 46% 5year event-free survival and 79% 5year overall survival. All had sensitive fusion tests giving high concordance between Ig/TCR and ABL-class fusion results (21 patients, *n* = 257 samples, r2 = 0.9786, *P* < 0.0001) and Ig/TCR and *IKZF1*-deletion results (9 patients, *n* = 143 samples, r2 = 0.9661, *P* < 0.0001). In contrast, in *BCR-ABL1* patients, Ig/TCR and *BCR-ABL1* tests were discordant in 32% (40 patients, *n* = 346 samples, r2 = 0.4703, *P* < 0.0001) and *IKZF1*-deletion results were closer to Ig/TCR (25 patients, *n* = 176, r2 = 0.8631, *P* < 0.0001).

**Conclusions:**

MRD monitoring based on patient-specific assays detecting gene fusions or recurrent assays for *IKZF1*-deletions is feasible and provides good alternatives to Ig/TCR tests to monitor MRD in ABL-class ALL.

## Introduction

ABL-class fusions are a feature of approximately 3% of paediatric acute lymphoblastic leukaemia (ALL) cases [1, 2] with similar gene expression patterns to Philadelphia chromosome positive (Ph-pos) ALL and with generally poor responses to standard induction chemotherapy. While Ph-pos ALL results from a t(9;22) translocation creating a *BCR-ABL1* fusion; this subset of Ph-like ALL cases involve the fusion of another gene expressed during lymphocyte differentiation such as *EBF1*, *SSBP2*, *ETV6*, *NUP214* with a gene encoding a tyrosine kinase or a receptor tyrosine kinase such as *PDGFRB*, *CSF1R*, *ABL1* or *ABL2*. ALL patients with these ABL-class fusions are generally sensitive to tyrosine kinase inhibitors (TKI) including dasatinib in vitro [3] and adjuvant TKIs in patients [4, 5]. Two recent studies from the AIEOP-BFM and Pont di Legno groups showed the 5-year event-free survival (EFS) for patients with ABL-class fusions in the pre-TKI era was 49% (*n* = 46) [1] and 59% (*n* = 122) respectively [2].

The poor outcomes associated with both *BCR-ABL1* ALL and Ph-like ALL mean that many treating clinicians request close MRD monitoring for these patients, during initial therapy and particularly for post-remission surveillance, including after HSCT. Moreover, in the TKI era, where HSCT is no longer indicated in many patients with *BCR-ABL1* ALL [6], accurate determination of post-treatment MRD is critical to identify patients with sub-optimal TKI response, where HSCT may still offer the best chance of cure. However, in a previous study of *BCR-ABL1* ALL [7], we demonstrated that MRD analysis using conventional MRD markers based on immunoglobulin and T-cell receptor (Ig/TCR) rearrangements fails to detect or underestimates MRD compared to qPCR genomic tests detecting the *BCR-ABL1* gene fusion itself in some CML-like patients. It is also accepted in *KMT2A*-rearranged infant ALL, that MRD testing based on detection of this disease related fusion is not only feasible, but also preferable to Ig/TCR markers given the high incidence of oligoclonality and earlier stage of cell of origin that characterises ALL with translocations of the KMT2A gene (previously known as MLL) [8, 9]. This collective knowledge raised the question of the reliability of immunoglobulin and T-cell receptor gene markers (Ig/TCR) in patients with ABL-class fusions.

Both *BCR-ABL1* ALL and Ph-like ALL have a high incidence of *IKZF1* deletions [10, 11]. Copy number analysis by microarray or by MLPA has revealed a variety of *IKZF1* deletions in ALL cases and their poor prognosis in newly diagnosed B-ALL was shown in German, Dutch, Italian and Australian cohorts [12–15]. International collaborations have included *IKZF1* deletions in multifactorial risk analysis [16, 17] and provided evidence that most if not all *IKZF1* deletions are associated with high risk of relapse [18] including recurrent internal deletions that are amenable to detection by generic qPCR MRD assays [19, 20]. These assays serve a dual purpose, with capacity to rapidly identify a subset of *IKZF1* high risk patients as well as to measure MRD without requiring prior sequencing.

This study therefore set out to develop and evaluate patient-specific qPCR MRD assays for paediatric ALL cases with *EBF1-PDGFRB, SSBP2-CSF1R*, *NUP214-ABL1* and other *ABL1* gene fusions and to compare the MRD results obtained with those based on *IKZF1*-deletion and conventional Ig/TCR qPCR MRD measurements. We used two different next generation sequencing (NGS) strategies - targeted and whole genome sequencing - to determine the precise breakpoint sequences needed to design patient-specific qPCR assays for ABL-class fusions.

## Methods

### Patient samples

This study was conducted on DNA samples from 65 paediatric ALL patients with parental consent and human ethics approval. All bone marrow samples were originally tested for MRD in response to clinical requests and results reported in real-time. The same samples were stored for research including retesting (with technical triplicates) to compare MRD levels obtained using alternate MRD assays.

### Identification of patients with ABL-class fusions

Patients with ABL-class fusions were provisionally identified by several methods: (a) G-banded karyotyping and fluorescent in situ hybridization as performed as standard of care with *BCR, ABL1* and *PDGFRB* at some centres; (b) MLPA analysis performed with SALSA P335 ALL-IKZF1 A4 or B1 kit (MRC-Holland, Amsterdam, the Netherlands) [15] since we discovered that patients with *EBF1-PDFGRB* have heterozygous loss of *EBF1* exon 16; (c) Ph-like TLDA expression pattern in unselected cohort [21] or (d) patients referred on basis of high risk features (defined by ANZCHOG 2014 guidelines). Fusion transcripts were analysed by RT-PCR and Sanger sequencing or RNA-Seq.

Following provisional identification, precise genomic breakpoints were identified in diagnostic DNA using multiplex long-distance PCR for *BCR-ABL1* [7] or by analysis of targeted NGS for difficult *BCR-ABL1* cases and 12 ABL-class cases.

### Analysis of breakpoint sequence for ABL-class fusions from WGS sequence

Two of the ABL-class cases were enroled on the PRISM precision medicine trial (NCT03336931) which used WGS analysis to at least 90x-depth of leukaemia cell DNA and 30x germline DNA [22]. The other seven ABL-class cases were sequenced using WGS to 30x coverage with no matched germline, reasoning that the somatic ABL-class fusions would be readily identifiable. WGS was conducted at the Kinghorn Centre for Clinical Genomics, Garvan Institute of Medical Research (Australia), using the Illumina HiSeq X Ten platform with a paired-end read length of 150 bases. Sequencing libraries were prepared from more than 1 µg of DNA using KAPA PCR-Free v2.1 (Roche). Raw fastq files were aligned to the hs37d5 reference genome using BWA-MEM (v0.17.10-r789) [23] with resulting BAM file reads marked using Novosort (v1.03.01; default settings). For cases with a matched germline, the WGS data were analysed as previously described [22]. For cases without a matched germline, a tumour-only analysis pipeline was adopted, using the following steps: somatic SNVs and short indels (<50 bp) were identified using Sage (v2.2) [24] and germline variants were filtered out using a panel of normals. The panel of normals contains variants identified from 1000 germline controls, sequenced using WGS to 30–40x depth using HiSeq X10, where each variant was observed at least once, with at least three reads and a cumulative base quality of 30. Somatic variants were annotated using SnpEff (v4_3t) [25] and imported into the in-house Glooee platform for filtration and prioritization. Tumour purity, ploidy and somatic copy number variants (CNVs) were identified using PURPLE (v3.0) [24], and structural variants (SVs) were identified using GRIDSS (v2.9.4) [26] and then annotated using Ensembl genes. LINX (v1.16) was used to visualize SV clusters and derivative chromosomes [27].

### MRD q-PCR assays to detect ABL fusions, IKZF1 deletions and Ig/TCR rearrangements

MRD tests for ABL-class fusions involved patient specific primers and a Taqman hydrolysis probe spanning the unique breakpoint sequence as reported previously for *BCR-ABL1* [7] and testing bone marrow DNA samples usually retrospectively. The unique breakpoint sequences for these patients and custom primers and probes are shown in Supplementary Tables 1 and 2.

Routine PCR-MRD marker screening was performed by 24 single or multiplex PCR reactions on leukaemic DNA to detect rearrangements in immunoglobulin heavy and kappa genes and T-cell receptor gamma, delta, beta and delta-alpha genes (Ig/TCR) followed by heteroduplex analysis and direct Sanger sequencing. Unique breakpoint sequences were identified using the NCBI Nucleotide BLAST database, https://blast.ncbi.nlm.nih.gov/ or hardcopies circulated by the EuroMRD group. The actual MRD testing involved q-PCR assays to detect these markers using one patient-specific primer and a gene segment specific primer and hydrolysis probe performed on an Biorad Icycler or CFX platform in real time [28].

Diagnostic samples for all patients were screened for four *IKZF*1 MRD markers to detect internal gene deletions specifically *IKZF1*Δ2-7, *IKZF1*Δ4-7, *IKZF1*Δ2-8, *IKZF1*Δ4-8, using generic qPCR tests with custom made primers and Locked Nucleic Acid (LNA) probes (Integrated DNA Technologies). For patients with a high level of marker (>1 × 10^−1^ level present in positive controls), the same assay was then used with the patient’s own dilution curve to measure MRD in their remission samples.

### Data analysis

All MRD qPCR tests, including the ABL-class fusion and *IKZF1* deletion assays, were performed at the Children’s Cancer Institute and analysed according to the guidelines established by EuroMRD (van der Velden et al, 2007). Standard curves met minimum standards of >0.98 correlation coefficient and slope between 3.1 and 3.9. MRD was scored positive or negative according to the definition established for protocols in which therapy intensification is intended and with reference to normal peripheral blood mononuclear cell DNA samples.

When comparing MRD levels determined by different tests concordant results were defined as those with <1.0 log difference in quantitative results or either negative or non-quantifiable results with both marker tests. In contrast discordant results had >1.0 log difference in quantifiable results. When one marker gave a quantifiable result and the other gave non-quantifiable or negative result, the quantitative range was considered. Results were defined as discordant if there was <1.0 log difference between the quantitative result and the quantitative range for the assay used for non-quantifiable positive or negative result. Survival times were measured from date of diagnosis to date of relapse or death, or to last clinic visit for patients without these events. Kaplan-Meier survival curves were generated and log-rank tests applied using GraphPad Prism version 7.04.

## Results

### Description of patients with ABL-class fusions and *BCR-ABL1*

**Fig. 1:**
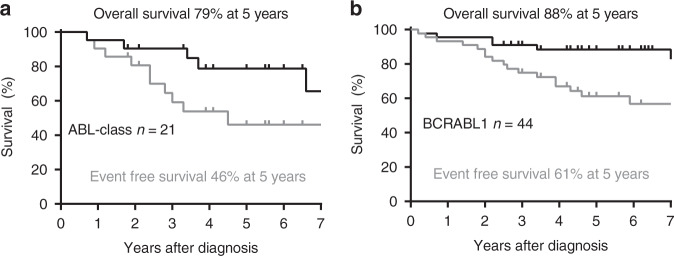
Outcomes for ALL patients included in this study. **a** Patients with ABL-class fusions including EBF1-PDGFRB and NUP214-ABL1 (**b**) patients with BCR-ABL1. The black line in each graph denotes overall survival and grey line event-free survival.

A database search of ALL patients with both qPCR MRD testing and research consent identified 21 paediatric patients with ABL-class fusions including 19 B-ALL and two T-ALL (Table 1). These patients were diagnosed between 2004 and 2018 with a median follow up of 5.5 years for survivors. We also identified an additional 44 children with *BCR-ABL1* ALL diagnosed in that timeframe (median follow up of 5.7 years) for comparison. The most frequently identified ABL-class fusions were *EBF1-PDGFRB* (9 cases, 43%) and *NUP214-ABL1* (4 cases, 19%). Two patients had *SSBP2-CSF1R*, two had other *PDGFRB* fusions and the remaining four patients had other *ABL1* fusions. The patients were predominantly male (71%) with median age of 10 years and 82% had high MRD at end of induction. In contrast to the others, the two patients with *ETV6-ABL1* fusion were both one year old females with complete MRD response (MRD negative) (Table 1). As a group, the ABL-class fusions had 5-year EFS of 46% and 5-year OS of 79%. While it appears from the Fig. 1a and b data, that outcomes may be poorer for patients with ABL-class compared to *BCR-ABL1*, these differences were not statistically significant. It is also worth noting that seven (33%) of the ABL-class patients did not receive a TKI (imatinib or dasatinib) in first remission, compared to one of 44 *BCR-ABL1* patients (Table 1).

**Table 1:** Characteristics of ALL patients included in ABL fusion marker study.

ID	ALL type	ABL Fusion	Recurrent IKZF1del	Sex	Age (years)	Front-line therapy	End of Induction MRD (d28-35)	Relapse/SMN (mo)	Later therapy	HSCT (stage)	Death (mo)	Current Status	Discordant MRD
1	B	EBF1-PDGFRB	IKZF1 4-7	M	5	BFM, no TKI	6 × 10^–2^	23	ALLR3, no TKI	CR1 + CR2		CR2	No
2	B	EBF1-PDGFRB	neg	F	11	BFM, no TKI	1 × 10–0 rel	37	ALLR3, no TKI	CR2	45	deceased	No
3	B	EBF1-PDGFRB	IKZF1 4-7	M	14	BFM, no TKI	1 × 10^–0^		–	CR1		CR1	No
4	B	EBF1-PDGFRB	neg	M	3	BFM, no TKI	1 × 10^–2^	30	ALLR3, imatinib	CR3		CR3	No
5	B	EBF1-PDGFRB	IKZF1 4-8	M	6	COG, imatinib	5 × 10^–2^	40	ALLR3, dasatinib	CR1 + CR2		CR2	No
6	B	EBF1-PDGFRB	neg	M	5	COG, imatinib	3 × 10^–3^	–	–	no		CR1	No
7	B	EBF1-PDGFRB	IKZF1 4-7	M	14	COG, imatinib, dasatinib	1 × 10^–1^	–	–	CR1		CR1	No
8	B	EBF1-PDGFRB	neg	M	7	BFM, imatinib	3 × 10^–1^		–	CR1		CR1	No
9	B	EBF1-PDGFRB	scIKZF1 4-7	F	15	COG, dasatinib	8 × 10^–2^	–		no		CR1	No
10	B	CD74-PDGFRB	IKZF1 4-7	F	2	COG, dasatinib	N/A	–	CAR-T, HSCT	CR1		CR1	No
11	B	AT7IP-PDGFRB	neg	M	9	BFM, dasatinib	7 × 10^–2^	11	blinatumomab, CAR-T	CR1 + CR2		CR3	No
12	B	ETV6-ABL1	neg	F	1	BFM, no TKI	neg	55	ALLR3, no TKI	CR1		CR2	No
13	B	ETV6-ABL1	scIKZF1 4-7	F	1	COG, no TKI	neg	–	–	no		CR1	No
14	B	IGSF11-ABL1	IKZF1 4-7	M	14	COG, dasatinib	1 × 10^–0^	–	vinc/steroid; HSCT	CR1		CR1	No
15	B	SNX2-ABL1	IKZF1 2-7	M	12	COG, dasatinib	3 × 10^–2^	–	blinatumomab, HSCT	CR1		CR1	No
16	B	NUP214-ABL1	IKZF1 4-7	M	16	BFM, dasatinib	9 × 10^–2^	–	–	CR1		CR1	No
17	B	NUP214-ABL1	IKZF1 4-7	M	12	COG, imatinib	4 × 10^–1^	–	blinatumomab	CR1		CR1	No
18	ETP	NUP214-ABL1	neg	M	10	COG, dasatinib	2 × 10^–2^	29 (SMN)	AML therapy	CR1 + CR2	41	deceased	No
19	T	NUP214-ABL1	neg	M	14	COG, dasatinib	7 × 10^–1^rel	15	IntReALL	CR2	21	deceased	No
20	B	SSBP2-CSF1R	neg	M	<1	BFM, no TKI	neg	34	ALLR3, no TKI	intent	79	deceased	No
21	B	SSBP2-CSF1R	neg	F	15	BFM, dasatinib	2 × 10^–2^	–	–	CR1	9	deceased	No
**Total**	**B-ALL**		**IKZF1del**	**Male**	**Age (median, range)**	**Front-line TKI** **use %**	**High EOI** **MRD > 1 × 10**^**–3**^ **(%)**	**Relapse or** **SMN**		**HSCT %**	**Death**	**Current CR1** **n, %**	**Discordant MRD**
*n* = 21	90%	ABL-class fusions	43%	71%	10 (0-16)	67%	85%	43%		81%	24%	52%	0%
*n* = 44	96%	BCR-ABL1	59%	59%	8 (0-17)	98%	66%	32%		68%	16%	61%	33%

Each of the 21 ABL-class patients are shown in the top section and then summarised for comparison with the *BCR-ABL1* patients in the bottom section. Patient 5 was included in Roberts et al. [3].

### Comparison of ABL-class fusion MRD tests with Ig/TCR tests

**Fig. 2:**
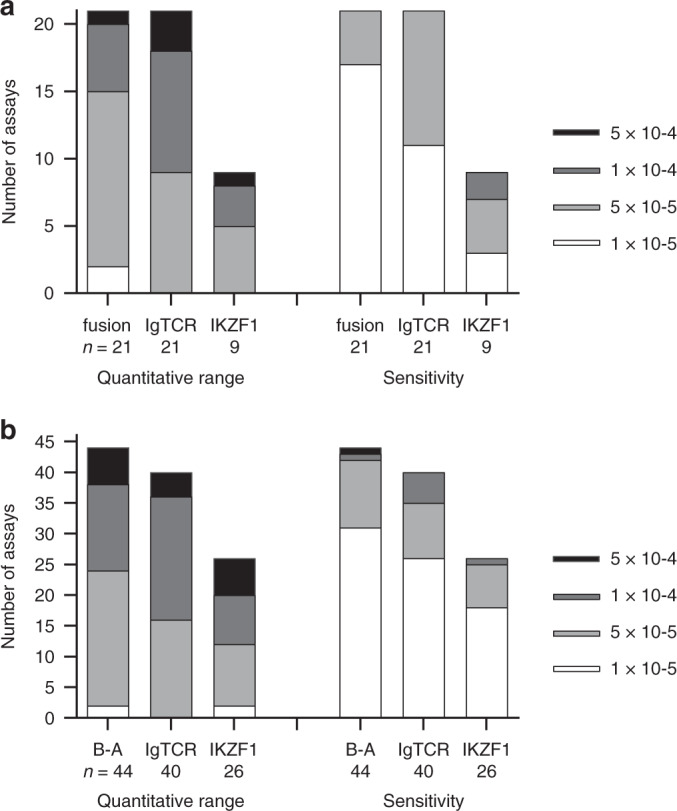
Evaluation of quantitative range and sensitivity of MRD assays specific for gene fusions, clonal Ig/TCR rearrangements and recurrent *IKZF1* deletions. **a** 21 patients with the ABL-class fusions shown in Table 1 and (**b**) 44 *BCR-ABL1* patients. B-A denotes data for *BCR-ABL1* fusion assays.

In order to determine precise genomic breakpoints and to design fusion-detecting qPCR MRD assays, targeted next generation sequencing (NGS) was performed on diagnostic bone marrow DNA from the 44 *BCR-ABL1* cases and 12 of the ABL-class patients. These included all cases with fusions involving *ABL1*, except one *NUP214-ABL1*, and five cases with *PDGFRB* fusions. For the remaining nine patients, whole genome sequencing (WGS) was performed and analysed for breakpoints including five with *EBF1-PDGFRB*, 1 *ATP7IP-PDGFRB*, 1 *NUP214-ABL1* and 2 *SSBP2-CSF1R*. These breakpoints were used to design patient-specific qPCR assays composed of two primers specific for each of the genes involved and a hydrolysis probe spanning the precise breakpoint sequence. Each assay was then evaluated for quantitative range and sensitivity according to the widely used EuroMRD guidelines that evaluate amplification of duplicates in a standard curve created from a dilution series of the diagnostic bone marrow DNA sample and 6 normal DNA samples from mononuclear cells. The analysis in Fig. 2a showed that the assays measuring the ABL-class fusion breakpoints had adequate sensitivity (1 × 10^-4^) in all and were highly sensitive in most patients (1 × 10^-5^ in 81%), with an acceptable quantitative range (QR at least 1 × 10^-4^) for all patients except one with QR of 5 × 10^-4^ and superior quantitation in 71% (QR of 5 × 10^-5^). These standardised MRD assay metrics compared favourably with conventional Ig/TCR based MRD tests in the same patients and same samples tested earlier and reported in diagnostic MRD reports (Fig. 2a).

**Fig. 3:**
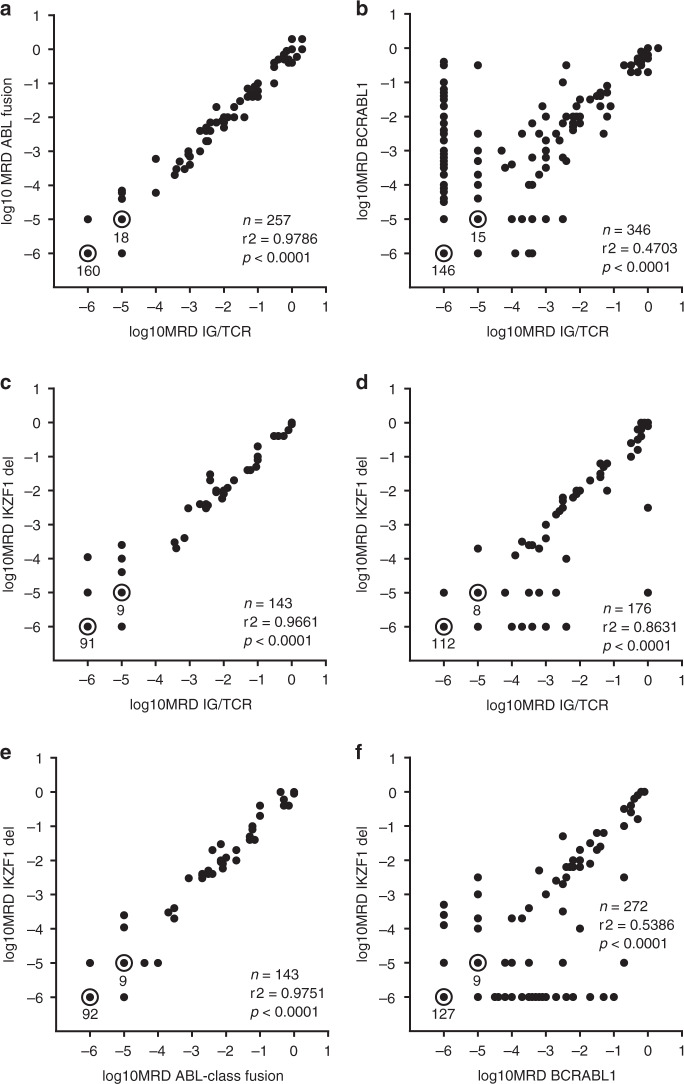
Relationships between MRD levels measured by quantitative real-time PCR on the same DNA samples with different types of MRD markers. **a** ABL fusion versus Ig/TCR tests for 21 patients. All assays were patient-specific using primers and probes shown in the supplement. Samples collected from Patient 18 after an AML secondary malignancy were excluded. **b** *BCR-ABL1* versus Ig/TCR tests for 40 patients. All assays were patient-specific. **c** *IKZF1* versus Ig/TCR tests in nine ABL-class patients. Three different IKZF1 generic tests were used. **d** *IKZF1* versus Ig/TCR tests in 23 *BCR-ABL1* patients. Four different *IKZF1* tests were used. **e** *IKZF1* versus ABL-class tests in nine ABL-class patients. **f** *IKZF1* versus *BCR-ABL1* tests in 26 *BCR-ABL1* patients.

These new MRD assays were then utilized to re-measure MRD levels in 257 bone marrow samples from the 21 ABL-class patients previously tested using Ig/TCR marker tests (Fig. 3a). The MRD data obtained were highly correlated with Ig/TCR results (Pearson correlation coefficient r2 of 0.9123, *P* < 0.0001). All of the patients had concordant results, defined by <1.0 log difference in quantifiable results or non-quantifiable or negative results for both samples.

A similar comparison was performed for 40 *BCR-ABL1* patients whose MRD had been evaluated with MRD assays designed to detect the genomic breakpoint as well as an Ig/TCR clonal marker (Fig. 3b). In contrast to ABL-class patients, 13 *BCR-ABL1* patients (33%) had discordant MRD results with higher MRD results obtained with the *BCR-ABL1* marker in 12 cases and higher Ig/TCR MRD levels in the remaining patient. These marked differences in some patients contributed to a much lower correlation coefficient for the results of the parallel MRD testing of the same samples using 2 different marker types in *BCR-ABL1* patients (40 cases, r2 = 0.4703, *P* < 0.0001, Fig. 3b).

### Comparison of *IKZF*1 MRD tests with Ig/TCR tests

**Fig. 4:**
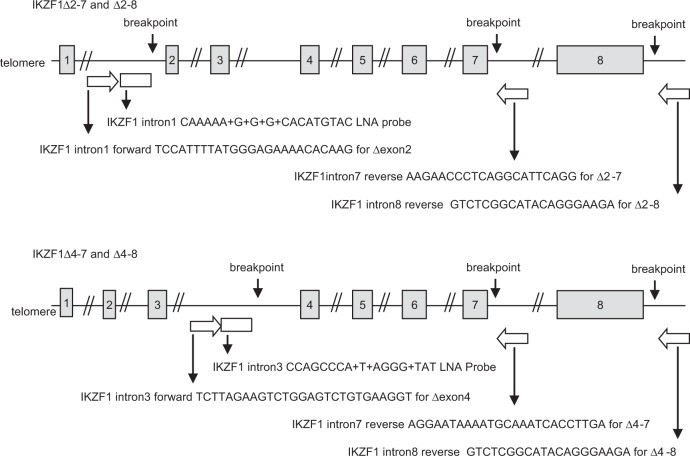
Sites and sequences of primers and probes used for q-PCR analysis to detect and measure recurrent deletions in the *IKZF1* gene. Each of the four assays requires a forward primer, a fluorescent Locked Nucleic Acid (LNA) hydrolysis probe and a reverse primer.

All ABL-class and *BCR-ABL1* patients were also screened with four qPCR assays designed to detect *IKZF*1Δ4-7, *IKZF*1Δ2-7, *IKZF*1Δ2-8 and *IKZF*1Δ4-8 respectively (Fig. 4). Of the 21 ABL-class fusion cases, nine (43%) showed a high level of at least one of these deletions gauged to be suitable for an effective MRD test because the level of deletion was greater than 1 × 10-1 compared to our positive control DNA for the deletion derived from primary patient cells or patient-derived xenograft. An additional two cases had a deletion at a sub-clonal level (Table 1). In comparison, screening for the four *IKZF*1 deletion assays identified 26 (59%) of the *BCR-ABL1* patients with a high level for one of these dual-purpose markers. The sensitivity and QR levels for these generic *IKZF*1 qPCR tests were assessed using a dilution series of each patient’s diagnostic DNA to create standard curves that were also used to measure the remission samples in the same assay. The sensitivity and QR levels for generic *IKZF*1 assays were similar to the Ig/TCR assays for both ALL subtypes (Fig. 2a, b).

When the MRD levels measured by *IKZF*1 markers were compared with the results of Ig/TCR assays for the same 143 samples from ABL-class patients there was a > 1 log difference for only a single sample (from Patient 14) Fig. 3c. For *BCR-ABL1* patients, three patients had discordant results with samples giving different MRD levels (>1 log difference). The overall correlation for MRD levels measured by the *IKZF*1 compared with Ig/TCR markers in ABL-class patients were better for patients with ABL-class fusions (r2 = 0.9661, Fig. 3c) than *BCR-ABL1* (r2 = 0.8631, Fig. 3d).

### Comparison of *IKZF1* MRD tests with fusion MRD tests

To improve our understanding of the variations in MRD level that are sometimes observed with different markers in particular patients, further comparisons were made between the MRD results obtained with the fusion-based versus *IKZF*1 deletion-based markers (Fig. 3e, f). The results of these two types of MRD tests on the same samples from patients with *BCR-ABL1* fusions showed relatively low Pearson’s correlation coefficient (0.5386, Fig. 3f). This reflects the observation that at the patient level, 6/26 (23%) patients had discordant MRD results. We were able to compare MRD determined by all three marker types for 23 *BCR-ABL1* patients –16 patients (70%) were concordant in all samples.

Again, higher concordance was observed for cases with ABL-class fusions compared to *BCR-ABL1* patients with none of the nine ABL-class patients showing discordant results with *IKZF1* markers (>1 log difference). In fact only 3 of 143 comparisons showed a difference >0.5 log (the 3 all from Patient 14) leading to a high Pearson’s coefficient (Fig. 3e, r2 = 0.9751, *P* < 0.0001).

## Discussion

This study demonstrated the feasibility of using targeted NGS or WGS to define genomic breakpoints and to design satisfactory qPCR MRD assays to monitor disease in ALL patients with ABL-class fusions. These 21 patients had poor outcomes, comparable to larger studies [1, 2], although we would expect this to improve with earlier diagnosis of the fusions and consistent intervention with TKIs [5]. The study also found a high incidence of recurrent *IKZF1* deletions in ABL-class patients consistent with a previous report of 40% *IKZF1* deletions in Ph-like ALL [11] and demonstrated effective use of these markers to monitor disease. Both the fusion and deletion MRD tests showed generally highly comparable results versus conventional Ig/TCR markers, thus providing additional scope to monitor MRD in these patients. MRD tests based on *IKZF1*-deletions were of particular interest since these deletions occurred in 23% of relapsed B-ALL [29] as well as Ph-like ALL and approximately half are recurrent and can be detected in multiple patients by generic assays [19, 20]. In this study, it was feasible to use these dual-purpose markers which do not require any prior sequencing to perform MRD testing in 43% of ABL-class patients and 59% of *BCR-ABL1* patients.

The analyses in this paper complement previous studies showing the potential to use genomic breakpoints for *BCR-ABL1* [7]; *KMT2A*-rearrangements [8, 9]; and *CDKN2A/B* deletions [30] as MRD markers in ALL. This is in line with our hypothesis that disease-drivers will make more reliable MRD markers than disease-passengers such as clonal Ig/TCR markers. While Ig/TCR markers have served as effective MRD markers for ALL, particularly when two markers are used as recommended by most trials [31], their use is restricted to lymphoid disease, they can underestimate MRD in *KMT2A*-rearranged infant ALL [9] and they can be subject to clonal evolution or selection at relapse [32].

However, each new genomic fusion or deletion marker should be considered on its own merits by careful evaluation and comparison with other methodologies before general diagnostic use for ALL or other diseases. In our laboratory, recurrent ALL genomic deletions in *BTLA* and *SLX4IP* genes did not appear to provide stable MRD markers (unpublished data). The approach used herein has the advantage that the well-established EuroMRD guidelines for evaluating the quantitative range and sensitivity of MRD tests in ALL can be readily applied to new genomic breakpoint assays and their MRD results, allowing fair comparisons to be made with measurement of Ig/TCR markers by either q-PCR [33] or by ddPCR [30].

Monitoring residual disease in patients with *BCR-ABL1* is challenging. Our previous study identified that a subset of ALL patients with *BCR-ABL1* and CML-like features had discordant MRD results measured by qRT-PCR or qPCR for *BCR-ABL1* and Ig/TCR [7]. This study used an extended series of *BCR-ABL1* patients and found discordancy in qPCR results of >1 log difference in 33% of patients between BCR-ABL1 and Ig/TCR compared to none of the 21 ABL-class ALL cases. The additional use of qPCR *IKZF1* deletion markers available in 59% of the *BCR-ABL1* patients did not progress our understanding although it served to illustrate further the inherent clonal instability in some of these patients. We have no definitive answer on the best way to monitor residual disease in *BCR-ABL1* ALL patients although the results of on-going trials such as EsPhALL trial will be informative.

Finally, as whole genome sequencing becomes more commonly used for ALL patients, this study shows it provides a viable alternative to multiple PCRs followed by heteroduplex analysis and Sanger sequencing for the detection of MRD markers in ALL. The ability to define the precise patient-specific genomic breakpoint sequences for key disease-related fusions and deletions that drive disease also suggests significant opportunity to develop in liquid biopsy assays for MRD in other cancers [30].

## Supplementary information

Supplementary Table 1

Supplementary Table 2

Checklist

## Data Availability

Data are securely stored at Children’s Cancer Institute with access limited by ethical considerations.
