# Supplementary material for: Measurable residual disease analysis in paediatric acute lymphoblastic leukaemia patients with ABL-class fusions
Source: Br J Cancer. 2022 Jun 1;127(5):908–15. doi: 10.1038/s41416-022-01806-6 (PMC9427854; doi:10.1038/s41416-022-01806-6)
Supplement: Supplementary file 1 — Supplementary Table 1 [file 41416_2022_1806_MOESM1_ESM.pdf]

Supplementary Table1 Breakpoint sequences for patients with ABL-class fusions

Patient		Fusion	Patient Breakpoint Sequences
1	A2489	EBF1-CDX1-PDGFRB	GCTGTGTTCTTGAGACTTGCTGGGGGCCTGGGGAAGGGGCATGGTTCCTGAGTCCCCAGAGTGTGATGTGTGATCT/GGG/AGCAAAGAAGCATAGATTGGGGACAGAAGAGGAGGGAATGAGTTCCATAGGACCTCATGGGTGGCCAGCAG
2	A3363	EBF1-PDGFRB	CTCAGAGCTGGTCTTCCACTCAGGCTGTCCACACTGTCTGCCCAGATACCCACTTGCTCAGAGAATGTGTATGCATGTGTGGGAGCATGGGTGAAATGAGGGGATGAAAGAAGCCCACCCATCCACCACAGGGCAGGGGACAGGAGGCAGCTCTAGACAGG/TTGTT/ACCTAAGGCTGGGTCCTCTTTGCCCTGCTGGAGATTGTGTGCAGAGGCTAAAGAGGCAAGTCCCAGAGTGTGGGCCCCACCTGCCAGCACAGCACCTGGGGGTGCAGTGGGCAGAAAGAAGAGCTTAAAGTGAGATGTTTACATTAATTTGACTTTAATTTCTCCATCCGTATGTCCATCAC
3	A5258	EBF1-PDGFRB	CCCTCCTCCCGCTCATCACACATCTCGGTCCTGGGAAGGAAGCCTGCATCTTCTAGGCAGCCAT/AACCCAGGGCCGAACA/GCAGTGAGTGAATCCCCTGGGTGCGTAAGTGAGGATGGAGGTGATGATGCCAAGGGCATGCCTGGGCAGT
4	A5491	EBF1-PDGFRB	GAATAAATGAAGCACACTCATACAGGTGCATGTATGCATAAGGACGGGCAGGTAGATACACAAAAGCATCAAGACACTGGTAGGCATTGTTCATAC//TGTTTTTTAAAAAAAGAAATAGCCAAGTTTATACATGATAGCACAAAATTGAAATTAAAAACAAATATATGGGTCTAGGGGGTGGGTTTGGCCCACTCC
5	A5901	EBF1-PDGFRB	ATTCATATCCATGGCTTATAGACTTGTGTTTGTACACGTGTGTACCCATATTGTGT/CCACCGCGCAAGGTGGAGGGGTCTATATTGTGTCG/GCCTGTGGCAAAAGGTGCAAGTCTACATTCGCTTTCCACCAAAATCTATTCTGCGTGCCT
6	A6788	PDGFRB/EBF1 (+strand)	CTGCCTCCCCAAGGATCTTTTCATCTGGGCTGGAGGTGGGTCCAGGCACCTTTATTAAAGACAGCGGGAGTTTTAAGCCTGTGGCAAAAGGTGCAAGTCTACTTTCGCTTTCCACCAAAATCTATTC/CCTGGGGAACCCTCCCCT/GTCACACAAAAGTGTCACATCTGCCTTGGCTCAGAGCTGGTCTTCCACTCAGGCTGTCCACACTGTCTGCCCAGATACCCACTTGCTCAGAGAATGTGTATGCATGTGTGGGAGCATGGGTGAAATGAGGGGATGAAAGAAGCCCACCCATCCACCACAGGGCAGGGGACAGGAGGCAGCTCTAGACAGGCACGTGCCTGAACATG
7	A7103	EBF1-PDGFRB	ACCCACTTGCTCAGAGAATGTGTATGCATGTGTGGGAGCATGGGTGA/GCACACACCAGACCCCACCCCTAACT/AAGTCAGAGCGAGATCAAGAGATTGTGAGAACACGGGTTATGACATTTTATAGATAAGGAGGCCAGTTCTCTGAGGT
8	A7360	EBF1-PDGFRB	ATTAGAAGGGGTCATTTTCCCCTGATGGCAATTGTTGCCTATGACCAAT/GGTTGCCTATGACCAACC/TTTTGTATTTTTAGTAGAAATGGGATTTCACCATATTGGCCAGGCCGGTCTTGAACTCCTGACCTCGTGATCCGCCCACCTTGGCCTCCCAAAGTGCTGGGATTACAGGCATGAGCCACTGCACCCAGCCTCCATCC
9	A7869	EBF1-PDGFRB	TAGCTTCTCTTGAAACATTTGAGAAGATCAGTGGCTGTGAGCAGGCATTCCTGCCCAGTGGCATTTGGTGGGAGCTGAGTCACTGCCCAGGCATGCCCTTGGCATCATCACCTCCATCCTCACTTACGCACCCAGGGGATTCACTCACTG/TAGGCCGTACTATTTGCATGTT/TAAGCCATGTACATGAGCATGCCTAATTCCCAGACATCTGTTTAAACCCCACAGTGTATGTCTGTATGCATGTACCGGTGGGTACACAATTCCTGTACATGTGTACAAGCAATGCATGTACAATTCCTTTGCATGCATGCAAACTGGCATAGTGCATGTCTGTAAGCATATAATTGGAGCAC
10	A8052	CD74-PDGFRB	CTTGCTGAGGTCCGAGGGTCTGGATGGTGAGTCCAGGGAGCATGAGAAATTCATCCAGGCCACACAGGCTCAGGCACCTATACTCCTGCCCACCCCCTGCAGGATGGATATTACCATTTGAAATACTTTGGCGCTTGCAGTGAGATCTTGCTTAAAAGAAGAAACCCACCAAATTCT//TTGGCAGAAGGTAAGCCCCTCCCTGGCCCAGTGGGTGAAGACTGGCACATCATTATTTGCATAAATTCCACCCCTCTGCCCTCCTTGAATGCTGCGTGATCTGGCCACATGCAGGGCTCAGCATGGAACTCAGGTGGGGACTTGTGACGCTGGCTGTCTCAGCTTCCTCCACCC
11	A7366	AT7IP-PDGFRB	TTACCATTCTGTGATTCCAACATTTTAAGCTTCCTGAATCAGGATTACAGCACCTCATGATCCCATCTTTCTA//GCTTCTGTTTTCTTTCTATAGTATCTTCAACCAATCTTGTCACTCCTCCAGCAGTTGTCAGTAGTCAACCTAAATT
12	A5047	ETV6-ABL1	GGGTTCAGGCGATTCTCCTGCCTCAGTGTCCTGAGTAGCTGGGATTACAGGCACACGCCACCATGCCTGGCTAAGTTTTTGTATTTTTAGTAGAGACGGGGTTTCGCCACGTTGGTCAGGCTGGTCTCAAACTCCTGGCCTCAAGTGATCCACCTGCCTCAGCTTCCCAAAGTGCTGGGATTACAGGCGTGAGCCACCACGCCCGGCCGTGTCT/ATTGGGGGTGGGAG/ACATAAAAGAGAAGAGATGTGGGACAATATAAATAGGCAGGAGACCAGGGTTGTAGACCCGGTACTATCACATTCCCAGCCTACCCCT
13	A6619	ETV6-ABL1	GAAAAATTCCAGGTGAGCCGTGGCAACAACCTCTAAAGGATCTAGGAACCTAAAGAGGATCTGCCTTTTATTTA//CCCTGATATCTCTTCAGGGGTTGAGAGGCTAAAACTATTTTCTTGTTAATAGAGTACCAAGGTCTTGTCTCTTTTGCTGTGTTGACGTTTGCAGTAATGGTACAAAAGTAAAGCAGGTGTCTTAGCATGATTC
14	A6224	IGSF11-ABL	AGTCCATTCTCACATTGCTATAAAGAACTACCTGAGTCTGGGTAATTTATAAAGAAAAGAGCTTCAGTTCGCTCACAGTTCTGCAGGATGTACAGAAAGCATGGCAGAGGAGGCCTCAGGAAACTTACAATCATGACAGAAAGTGAAGAGGAAGCAGACATGTCCTACATGGCTAGAGAAGGAGGAAGAGAA//CTAGAGGCAATCACCCTGGAGTTTTATGTGTGTCCTTCTAGAAATACTGTGTGCACATATAAGCATTTATTATAACATGTGAATTTTGCTCTCCAGCTTTTTGTGAATATGCATGTTTATAGCAG
15	A7328	ABL-SNX2	AGCTTCAGCAGAGTTGCAGGAAGGGACCATTTACACAGCCTCTTAGATGTTTAAGGTTTACCCGCCCTGACAGTGGCTCAGCAGGCGTAGGCCTCCTGGTGAAAAGCCGAGCACATGGCTAACCTGCTCTGGTGGAGGGGCTTTGGACCGTTCTTGTGTA/CCCCCCCC/CAGTGAGCTGAGATTGAGCCACTGCACTCCTGCCTGGGCAGCAGAGCAAGACTCTATCTTTAAAAAAAAAAAAAAAAAAAAATTGTTACTTCTCCAAATGGTCTTTTTCGTGTGTGTGTGTGTGTGTGTGTGTGTGTGTGTGTGTGTTTTA
16	A6382	NUP214-ABL	CAAAAAATGTTAAAATTCGCCAGACATGGTGGCACACACCTGTGGTCCCAGCTACTCGGGAGGCTGAAGCAGGAGGATCACTTGAGCGCTGCAGTGAGCTGTGATTGCACCACTGCACTCCAGCCTGGGTGACAGAATGAGACCCTGTCTCAAAATAAATAAATATGTAACATACTGAAAGTGGTTGAAACATATATATATATATATATTTTTGAGACGGAGTCTCGCTCTGTTGCCACCC/T/TTTGCTCGTTAAAGGAGCAGCTTTGAAATCTGGACTGCAGGGATATCCAAAACAACAACTGCATGTTTCTAAGGGA
17	A6919	NUP214-ABL	CTTGTGTTGTGATTACATAAACAATAAGAAGTCTGGATTTCATCCCTCTAACAACAGATTGACTTTCTGAAAGGGGAGAAAAGGCTTGTCGCTAGGCCACCATTACTCCCGCTAGCACGCAGTTCATAGGCCAACACTCACCTTAAGAAGTACTCCCTTTAGAGGGTGCTCCCAGTTAGAGCAC//GACTTCTCATACACTTATAAATGCATATCTTTATGTGGACTTGTTAAAATGAAATTGGTATTTAGGAATTTGGAGATTTTTAGTAGTTACACAAGAATCAATGAAAAAGAACGAAGCTGGTTTCCAAAGCTGATATG
18	A7236	NUP214-ABL	ATTTGGAAACAGTGGGGCCAAGACATTTGGTGGATTTGCCAGCTCGTCGTTTGGAGAGCAGAAACCCACTGGCACTTTCAGCTCTGGAGGAGGAAGTGTGGCATCCCAAGGCTTTGGGTTTTCCTCTCCAAACAAAACAGGTACTCCTATGTCTATTTGTTATGGTTATCTTTATATATGTATTTGTTTATGCATAGATATCTGGAAGGAGACA/GG/GGCTTTCCTCAGCAGTCTGGTAATCCTGGTTGTCCATTCATGCTTAAGAACATGGGAATGGAAGGGGTGTTGGGAGTCCTATATATGTGGATGGGGTTGTCCA
19	A7830	NUP214-ABL	ACCATGCCCAGCCCCCAAATCACTTTATTTTTAATTGAAGTATATGTTTATCCACAATCCACATAAGTACACAGCTGAATTAATTAT//ACTCAGTCTCCTGAGTAGCTGGGATTACAGGTACCCGCCACTAATTTTCATATTTTTGCCTGG
20	A4852	SSBP2-CSF1R	GTGCCTGCAGCTGGGCTCCTTCCCTGGGCACCCGAGCTAGAAACTTAAAGGAAGTGG/ACCCCCGAGGGAGTTCGAC/TATGGTGCAACAGGCACTAATGGTACTATCACCCAGGGGTCGGCCAGCTTAGAGCCCAGAGCCAATGAACCTGG
21	A6464	SSBP2-CSF1R	AACTTCCAGGCTCTGATTTGGAAGGGCGGACTGCAGGTGAACTTCAGAGCCTCTGCGTTATAGCTTGGG/GCTCC/CTTCAAGATAAAACCTGACAGATCCTTAAAAGGCAGACTGCTCAAAAATATGTGCTTTCAGAGTTATGTGTATTAAA
