# Supplementary material for: Measurable residual disease analysis in paediatric acute lymphoblastic leukaemia patients with ABL-class fusions
Source: Br J Cancer. 2022 Jun 1;127(5):908–15. doi: 10.1038/s41416-022-01806-6 (PMC9427854; doi:10.1038/s41416-022-01806-6)
Supplement: Supplementary file 2 — Supplementary Table 2 [file 41416_2022_1806_MOESM2_ESM.pdf]

Supplementary Table2 Patient-specific primers and probles for ABL fusion qPCR

Patient		Fusion	Forward Primer	Reverse Primer	Probe
1	A2489	EBF1-CDX1-PDGFRB	TGG TTC CTG AGT CCC CAG AGT	CCT CCT CTT CTG TCC CCA ATC T	TG ATG TGT GA TCT GGG AGC AAA GAA GCA
2	A3363	EBF1-PDGFRB	TGTGGGAGCATGGGTGAAAT	CTCTTTAGCCTCTGCACACAATCTC	AGCTCTAGACAGGTTGTTACCTAAGGCTGGGTCCT
3	A5258	EBF1-PDGFRB	GGA AGG AAG CCT GCA TCT TCT	CAT CAC CTC CAT CCT CAC TTA CG	CA GCC ATA AC CCA GGG CCG AAC A
4	A5491	EBF1-PDGFRB	AGGTAGATACACAAAAGCATCAAGACA	CCCCTAGACCCATATATTTGTTTTTAAT	TGGTAGGCATTGTTCATACTGTTTTTTTAAAAAAAGAAATAGC
5	A5901	EBF1-PDGFRB	TGT CCA CCG CGC AAG G	TTG GTG GAA AGC GAA TGT AGA CTT	TT GTG TCG GC CTG TGG CAA AAG GT
6	A6788	PDGFRB/EBF1 (+strand)	TCT ACT TTC GCT TTC CAC CAA AA	AGC CAA GGC AGA TGT GAC ACT T	AT TCC CTG GG GAA CCC TCC CCT GT
7	A7103	EBF1-PDGFRB	TTCCTCAACGGCTCAGCTG	CCACCAGGGCCAGGATG	CCCCCTATGCCACCTTGCCCTTTAA
8	A7360	EBF1-PDGFRB	TTCCCCTGATGGCAATTGTT	GACCGGCCTGGCCAA	CCAATGGTTGCCTATGACCAACCTTTTGTATT
9	A7869	EBF1-PDGFRB	CAC CTC CAT CCT CAC TTA CGC A	GCA TAC AGA CAT ACA CTG TGG GGT TTA	AT TCA CTC AC TGT AGG CCG TAC TAT TTG CAT GTT TAA GC
10	A8052	CD74-PDGFRB	GCT TGC AGT GAC ATC TTG CTT AAA	TGC AAA ATA ATG ATG TGC CAG TCT	AC CCA CCA AA TTC TTT GGC AGA AGG TAA GCC
11	A7366	AT7IP-PDGFRB	AACATTTTAAGCTTCCTGAATCAGGAT	ACAACTGCTGGAGGAGTGACAAG	ACCTCATGATCCCATCTTTCTAGCTTCTGTTTTCTTTC
12	A5047	ETV6-ABL1	TGC CTC AGC TTC CCA AAG TG	AAC CCT GGT CTC CTG CCT ATT T	CC GTG TCT AT TGG GGG TGG GAG ACA TAA
13	A6619	ETV6-ABL1	CGT GGC AAC AAC CTC TAA AGG	CAA CAC AGC AAA AGA GAC AAG ACC T	AG GAT CTG CC TTT TAT TTA CCC TGA TAT CTC TTC AGG
14	A6224	IGSF11-ABL	CAGAAAGTGAAGAGGAAGCAGACA	CTGGAGAGCAAAATTCACATGTTATAA	AAGGAGGAAGAGAACTAGAGGCAATCACCCT
15	A7328	ABL-SNX2	ACATGGCTAACCTGCTCT	GTGCAGTGGCTCAATCTC	TCTTGTGTACCCCCCCCAGTG
16	A6382	NUP214-ABL	GAC AGA ATG AGA CCC TGT CTC AAA	CCA GAT TTC AAA GCT GCT CCT T	CG CTC TGT TG CCA CCC TTT TGC TC
17	A6919	NUP214-ABL	AAC ACT CAC CTT AAG AAG TAC TCC CTT T	TGT GTA ACT ACT AAA AAT CTC CAA ATT CCT	CT CCC AGT TA GAG CAC GAC TTC TCA TAC ACT TAT AAA TG
18	A7236	NUP214-ABL	GGT ACT CCT ATG TCT ATT TGT TAT GGT TAT CTT TA	TCC ATT CCC ATG TTC TTA AGC AT	TC TGG AAG GA GAC AGG GGC TTT CCT CA
19	A7830	NUP214-ABL	CCAGCCCCCAATCACTTTA	ATGAAAATTAGTGGCGGGTACCT	ACACAGCTGAATTAATTATACTCAGTCTCCTGAGTAGCTGG
20	A4852	SSBP2-CSF1R	GGC ACC CGA GCT AGA AAC TTA AA	GGG TGA TAG TAC CAT TAG TGC CTG TT	AG TGG ACC CC CGA GGG AGT TCG AC
21	A6464	SSBP2-CSF1R	GGC GGA CTG CAG GTG AAC T	ACA TAT TTT TGA GCA GTC TGC CTT TT	TG CGT TAT AG CTT GGG GCT CCC TTC AAG ATA A
